# Is there an added value of faecal calprotectin and haemoglobin in the diagnostic work-up for primary care patients suspected of significant colorectal disease? A cross-sectional diagnostic study

**DOI:** 10.1186/s12916-016-0684-5

**Published:** 2016-09-26

**Authors:** Sjoerd G. Elias, Liselotte Kok, Niek J. de Wit, Ben J. M Witteman, Jelle G. Goedhard, Mariëlle J. L. Romberg-Camps, Jean W. M. Muris, Karel G. M. Moons

**Affiliations:** 1 Julius Center for Health Sciences and Primary Care, University Medical Center Utrecht, Stratenum 6.131, P.O. Box 85500, 3508 GA Utrecht, The Netherlands; 2 Department of Gastroenterology, Gelderse Vallei Hospital, Ede, The Netherlands; 3 Department of Gastroenterology, Atrium Medical Center, Heerlen, The Netherlands; 4 Department of Gastroenterology, Orbis Medical Center, Sittard, The Netherlands; 5 The Department of Family Medicine, Care and Public Health Research Institute (Caphri), Maastricht University, Maastricht, The Netherlands

## Abstract

**Background:**

The majority of primary care patients referred for bowel endoscopy do not have significant colorectal disease (SCD), and are – in hindsight – unnecessarily exposed to a small but realistic risk of severe endoscopy-associated complications. We developed a diagnostic strategy to better exclude SCD in these patients and evaluated the value of adding a faecal calprotectin point-of-care (POC) and/or a POC faecal immunochemical test for haemoglobin (FIT) to routine clinical information.

**Methods:**

We used data from a prospective diagnostic study in SCD-suspected patients from 266 Dutch primary care practices referred for endoscopy to develop a diagnostic model for SCD with routine clinical information, which we extended with faecal calprotectin POC (quantitatively in μg/g faeces) and/or POC FIT results (qualitatively with a 6 μg/g faeces detection limit). We defined SCD as colorectal cancer (CRC), inflammatory bowel disease, diverticulitis, or advanced adenoma (>1 cm).

**Results:**

Of 810 patients, 141 (17.4 %) had SCD. A diagnostic model with routine clinical data discriminated between patients with and without SCD with an area under the receiver operating characteristic curve (AUC) of 0.741 (95 % CI, 0.694–0.789). This AUC increased to 0.763 (95 % CI, 0.718–0.809; *P* = 0.078) when adding the calprotectin POC test, to 0.831 (95 % CI, 0.791–0.872; *P* < 0.001) when adding the POC FIT, and to 0.837 (95 % CI, 0.798–0.876; *P* < 0.001) upon combined extension. At a ≥ 5.0 % SCD probability threshold for endoscopy referral, 30.4 % of the patients tested negative based on this combined POC-tests extended model (95 % CI, 25.7–35.3 %), with 96.4 % negative predictive value (95 % CI, 93.1–98.2 %) and 93.7 % sensitivity (95 % CI, 88.2–96.8 %). Excluding the calprotectin POC test from this model still yielded 30.1 % test negatives (95 % CI, 24.7–35.6 %) and 96.0 % negative predictive value (95 % CI, 92.6–97.9 %), with 93.0 % sensitivity (95 % CI, 87.4–96.4 %).

**Conclusions:**

FIT – and to a much lesser extent calprotectin – POC testing showed incremental value for SCD diagnosis beyond standard clinical information. A diagnostic strategy with routine clinical data and a POC FIT test may safely rule out SCD and prevent unnecessary endoscopy referral in approximately one third of SCD-suspected primary care patients.

Please see related article: http://bmcmedicine.biomedcentral.com/articles/10.1186/s12916-016-0694-3.

**Electronic supplementary material:**

The online version of this article (doi:10.1186/s12916-016-0684-5) contains supplementary material, which is available to authorized users.

## Background

Patients with persistent lower abdominal complaints are common in primary care [1]. At presentation, the general practitioner (GP) has to differentiate between potentially life-threatening significant colorectal diseases (SCD), such as colorectal cancer (CRC) and inflammatory bowel disease (IBD), and functional bowel disorders such as irritable bowel syndrome. As symptoms and signs alone have insufficient specificity, GPs refer many patients for endoscopy to not miss an SCD diagnosis. Consequently, 60–80 % of referred patients do not have SCD at endoscopy [2–6], unnecessarily straining healthcare budgets and endoscopy schedules, and exposing many non-SCD patients to a small but realistic risk of severe endoscopy-associated complications.

Thus, an improved diagnostic strategy that can safely rule out SCD is needed. Previous – largely non-primary care – studies have shown that diagnostic strategies solely based on symptoms and signs are unlikely to suffice [7, 8]. Adding faecal biomarkers to such diagnostic strategies may, however, improve their performance. One promising faecal biomarker is calprotectin, which indicates the presence of intestinal inflammation [9]. Calprotectin has been recommended by the National Institute for Health and Care Excellence (NICE) to help distinguish between IBD and non-IBD [10]. However, calprotectin has only been evaluated as a single test without accounting for other diagnostic information [11–13]. Furthermore, the presence of faecal haemoglobin (Hb) may indicate neoplastic disease [14]. Faecal occult blood tests have previously been included in diagnostic strategies for CRC with limited success [15, 16]. Over the past decade these tests have improved substantially, mainly because of specific immunochemical detection of human Hb, resulting in so-called faecal immunochemical tests for Hb (FITs) [14].

We designed the large-scale prospective CEDAR study (Cost-Effectiveness of a Decision rule for Abdominal complaints in primary caRe), to develop a new diagnostic strategy to safely rule out SCD in primary care patients with lower abdominal complaints, thus reducing the number of unnecessary endoscopy referrals. To meet this aim, we specifically quantified the incremental diagnostic accuracy of a point-of-care (POC) calprotectin test and a POC FIT above routine diagnostic information, both individually and in combination. We specifically focused on POC tests as these can be easily executed at the time and place of patient care.

## Methods

### Study design

The prospective diagnostic CEDAR study enrolled patients from 266 Dutch primary care practices referred for endoscopy from July 2009 through January 2012 [11]. Patients were eligible if suspected of SCD, defined by lower abdominal complaints for at least 2 weeks, combined with rectal bleeding, change in bowel habit, abdominal pain, fever, diarrhoea, weight loss, and/or a sudden onset of abdominal complaints at > 50 years of age. Patients were excluded if aged below 18, known with SCD, or with confirmed parasitic bowel infection. Recruitment was at the GP’s office (19.0 %) or directly following endoscopy scheduling (81.0 %). If not directly recruited by their GP, our research staff contacted eligible patients. If at any time during the study patient referral outpaced our study resources, each n^th^ scheduled patient was screened and contacted to guarantee representativeness of the study population. The University Medical Center Utrecht ethics committee approved the study (protocol number 08-462E), and all patients gave written informed consent.

### History taking and physical examination

Patient and GP questionnaires facilitated a structured history taking. Abdominal pain, rectal blood loss or mucus, weight loss, and fever were considered present upon patient or GP report; duration of abdominal pain, abdominal bloating, and family history of CRC upon patient report; and change in bowel habit upon GP report. We defined constipation as at least two of the following symptoms: less than three bowel movements/week, difficult/incomplete defecation, hard/lumpy faeces, sensation of anorectal obstruction, or laxative use. We based diarrhoea on frequently loose/liquid faeces, or anti-diarrhoea medication use. GPs reported the presence of a palpable abdominal mass or an abnormal digital rectal examination.

### Blood and faecal SCD biomarkers

A pre-endoscopy venous blood sample was drawn to estimate Hb and C-reactive protein (CRP) concentrations according to routine clinical practice. Directly following study inclusion, patients provided faeces samples collected before bowel preparation for endoscopy in a plain blue-capped faecal container, and kept refrigerated (4 °C) for a maximum of 2 days before handing in. Study protocol allowed freezing (–20 °C) of faecal samples before processing (this occurred in 67.9 % of samples; median days between collection and processing: 10; 10th–90th percentile: 4–21). If not frozen, the refrigerated faecal samples needed to be processed for calprotectin testing within 6 days (adherence 96.3 %; median days: 2: 10^th^–90^th^ percentile: 0–3), and needed to be tested for Hb within 3 days of collection (adherence 94.5 %; median days: 2: 10^th^–90^th^ percentile: 0–3).

We analysed the faecal samples for calprotectin concentration by a quantitative POC test (Quantum Blue®; dynamic range 30–300 μg/g) and by an enzyme-linked immunosorbent assay (ELISA; EK-CAL Calprotectin ELISA, both from Bühlmann Laboratories), both yielding estimates of μg calprotectin/g faeces, and for faecal Hb by a qualitative POC FIT (Clearview® iFOBT One Step Faecal Occult Blood Test Device, Alere Health), yielding either a positive or negative test result (lower detection limit of 6 μg/g). Laboratory technicians performed the ELISA, and trained research nurses the POC tests, blinded for clinical information and according to the manufacturers’ instructions. Briefly, for the calprotectin assays, 80 mg homogenized faeces was centrifuged and the supernatant was tested for calprotectin (1:16 diluted for the POC test and undiluted for the ELISA; supernatant for the ELISA was stored at –20 °C for maximally 4 months before analysis); for the POC FIT three separate random areas of the faecal sample were stabbed by the specimen collection stick and transferred to the collection tube, and two drops of extracted specimen were then applied to the test device. For more details see Kok et al. [11].

### Diagnostic outcome

Experienced gastroenterologists from three high-volume centres (i.e. > 1000 endoscopies annually) performed endoscopy in all patients, i.e. colonoscopy or sigmoidoscopy. A final diagnosis was established according to routine clinical practice, including histopathology of biopsies if required, and 3 months follow-up after negative endoscopy. We defined SCD as CRC, IBD, diverticulitis, or advanced adenoma (AA; > 1 cm). Outcome assessment was blinded for the biomarker test results and other diagnostic information.

### Statistical analysis

In view of the number of SCD diagnoses [17], we first developed a basic diagnostic model for SCD considering 15 patient history and physical examination predictors (listed in Table 1) and simple blood analyses (Hb and CRP concentrations). We started by selecting patient history and physical examination predictors using Akaike’s Information Criterion (AIC)-based stepwise-backward logistic regression; first considering and selecting only the patient history predictors, and then considering and selecting the physical examination predictors while keeping the selected patient history predictors fixed. Subsequently, Hb and/or CRP were only selected if they significantly improved the patient history/physical examination model. We deliberately used a more stringent selection criterion for the blood analyses (*P* < 0.05 instead of AIC-based) in view of the patient burden associated with obtaining this information. Blood Hb and CRP were modelled continuously instead of using a threshold for abnormal values (e.g. defining anaemia), to preserve as much diagnostic information as possible.

**Table 1:** Distribution and accuracy of individual predictors for diagnosing SCD in primary care as observed in 810 Dutch patients with lower abdominal complaints referred for endoscopy in the CEDAR study^a^

		Diagnostic accuracy for SCD					
		SCD (n = 141; 17.4 %)		No SCD (n = 669; 82.6 %)			
Diagnostic predictor^b^	Distribution in CEDAR study, %^c^	Sensitivity, % (95 % CI)	PPV, % (95 % CI)	Specificity, % (95 % CI)	NPV, % (95 % CI)	AUC (95 % CI)	Percent missing
Patient history							
Male sex	45.1	48.2 (40.1–56.4)	18.6 (15.0–22.9)	55.6 (51.8–59.3)	83.6 (79.9–86.7)	–	0.0
Age in years, median (min–max)	61 (19–92)	–	–	–	–	0.602 (0.553–0.652)	0.0
Absence of abdominal pain	19.3	33.0 (25.7–41.1)	29.7 (23.1–37.3)	83.5 (80.5–86.1)	85.5 (82.6–88.0)	–	0.4
Duration of abdominal pain in weeks, median (min–max)	90 (0–10 years)^d^	–	–	–	–	0.597 (0.543–0.652)^e^	7.4
Rectal blood loss	43.6	66.0 (57.8–73.3)	26.3 (22.0–31.1)	61.1 (57.3–64.7)	89.5 (86.3–92.0)	–	0.6
Rectal mucus	38.0	51.3 (43.1–59.5)	23.5 (19.1–28.6)	64.9 (61.2–68.4)	86.3 (83.1–89.1)	–	1.2
Weight loss	19.2	23.8 (17.4–31.5)	21.6 (15.8–28.7)	81.8 (78.7–84.6)	83.6 (80.5–86.2)	–	0.7
Change in bowel habit	65.5	68.0 (58.6–76.4)	18.1 (14.9–21.7)	35.1 (31.4–38.9)	83.9 (78.8–88.1)	–	12.3
Absence of abdominal bloating	45.0	60.5 (52.1–68.4)	23.4 (19.3–28.1)	58.3 (54.5–62.1)	87.5 (84.1–90.3)	–	4.2
Absence of fever	89.0	89.2 (83.0–93.4)	17.4 (14.8–20.4)	11.0 (8.9–13.6)	82.9 (73.7–89.4)	–	0.6
Absence of constipation	42.1	50.0 (41.8–58.2)	20.7 (16.7–25.3)	59.6 (55.7–63.3)	85.0 (81.4–87.9)	–	3.5
Diarrhoea	29.1	29.4 (22.4–37.4)	17.6 (13.2–23.0)	71.0 (67.4–74.3)	82.7 (79.3–85.5)	–	1.6
Absence of family history of CRC	81.7	84.3 (76.0–90.6)	17.9 (15.1–21.1)	18.8 (15.7–22.3)	85.0 (77.6–90.7)	–	14.2
Physical examination							
Absence of palpable abdominal mass	95.2	95.6 (89.5–99.3)	17.5 (14.9–20.4)	4.9 (3.4–6.9)	84.4 (67.3–95.3)	–	14.8
Abnormal digital rectal examination	7.8	11.5 (6.6–18.5)	25.7 (14.5–39.7)	92.9 (89.8–95.5)	83.3 (80.4–85.8)	–	17.7
Blood analyses							
Hb in g/dL, mean (SD)	14.2 (1.3)	–	–	–	–	0.556 (0.502–0.610)^f^	4.4
Anaemia (♀ < 12 and ♂ < 13 g/dL)	5.5	9.9 (5.8–16.0)	31.0 (18.9–46.1)	95.4 (93.4–96.8)	83.4 (80.6–85.9)	–	4.4
CRP in mg/L, median (min–max)	2 (0–20)^d^	–	–	–	–	0.587 (0.535–0.638)	4.6
Elevated CRP (≥10 mg/L)	9.4	12.2 (7.5–18.9)	22.7 (14.5–33.5)	91.2 (88.8–93.2)	83.1 (80.2–85.7)	–	4.6
Faecal tests							
Calprotectin POC test in μg/g, median (min–max)	48 (30–300)	–	–	–	–	0.681 (0.629–0.732)	5.6
Positive calprotectin POC test (>50 μg/g^g^)	48.7	69.6 (61.4–76.7)	24.9 (20.8–29.4)	55.7 (51.8–59.5)	89.7 (86.4–92.3)	–	5.6
Calprotectin ELISA test in μg/g, median (min–max)	62 (8–500)^d^	–	–	–	–	0.661 (0.606–0.716)	8.6
Positive calprotectin ELISA test (>50 μg/g^g^)	56.6	71.4 (63.2–78.5)	21.9 (18.4–26.0)	46.5 (42.6–50.4)	88.5 (84.7–91.5)	–	8.6
Positive POC FIT (>6 μg Hb/g^h^)	25.1	67.2 (58.9–74.5)	46.5 (39.6–53.7)	83.7 (80.5–86.6)	92.4 (89.9–94.3)	–	6.2

*AUC* area under the receiver operating characteristic curve; *CEDAR* Cost-Effectiveness of a Decision rule for Abdominal complaints in primary caRe; *CI* confidence interval; *CRC* colorectal cancer; *CRP* C-reactive protein; *ELISA* enzyme-linked immunosorbent assay; *FIT* faecal immunochemical test for haemoglobin; *Hb* haemoglobin; *NPV* negative predictive value; *POC* point-of-care; *PPV* positive predictive value; *SCD* significant colorectal disease; *SD* standard deviation

^a^Distribution and accuracy estimates per diagnostic predictor are following multiple imputation of missing values

^b^Predictors are coded such that the reported category indicates a higher risk of SCD, to allow direct comparison of accuracy measures across predictors

^c^If not otherwise stated

^d^Outliers were truncated

^e^Negative relation with presence of SCD

^f^U-shape relation with presence of SCD

^g^Manufacturer’s threshold

^h^Lower detection limit as stated by manufacturer

We then added the faecal biomarker tests to this basic diagnostic model (the calprotectin tests continuously and the POC FIT dichotomously), resulting in five extended models: three separate extensions (calprotectin POC or ELISA, or POC FIT), and two combined extensions (calprotectin POC or ELISA with POC FIT). As faecal testing may also be burdensome, we used the same stringent selection criterion for each faecal biomarker test as for the blood analyses (i.e. *P* < 0.05 for model improvement). Any blood analysis included in these extended models was subsequently removed if non-significant. For those models extended with the FIT, we also considered whether the FIT diagnostic odds ratio for SCD was lower in patients with overt rectal blood loss compared to those without (implying less diagnostic information), by testing a [FIT*blood loss] interaction term. All predictor selection tests were based on the log likelihood ratio. In all modelling, continuous predictors were included as such, using transformations if necessary to maintain linearity, while truncating outliers. Transformations were necessary for blood Hb (U-shape relation with SCD risk), and for duration of abdominal pain and CRP (logarithmic relations). See Additional file 1 for further model development details.

The final six diagnostic models were assessed for discrimination (area under the receiver operating characteristic curve; AUC), calibration, explained variation (Nagelkerke R^2^), accuracy (i.e. sensitivity, specificity, negative and positive predictive values (NPV and PPV) at different SCD probability thresholds: 2.5 %, 5.0 % and 7.5 %), and net benefit (decision curve analysis) [18–20]. All faecal biomarker extended models were compared to the basic model and the combined biomarker extended models to the individual biomarker extended models, in terms of discrimination, explained variation, and reclassification (net reclassification improvement (NRI) at 5.0 % and 50.0 % probability threshold for low and high risk, and (relative) integrated discrimination improvement (IDI)) [21].

We used 500-fold bootstrap resampling, including predictor selection, to derive optimism-corrected AUCs, Nagelkerke R^2^s, and regression coefficients [22]. We multiple imputed the 5.2 % missing data points [23–25], and pooled the results from the 10 imputed datasets [26, 27]. Analyses were performed in R version 3.1.3. All *P* values are two-sided. This publication adheres to the TRIPOD statement [28].

## Results

### Study population

**Fig. 1:**
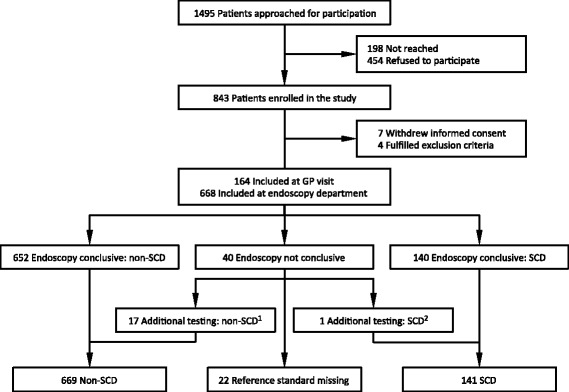
Flowchart of Dutch primary care patients with lower abdominal complaints for at least 2 weeks and referred for endoscopy, and their enrolment in the CEDAR study from July 2009 through January 2012. *CEDAR* Cost-Effectiveness of a Decision rule for Abdominal complaints in primary care; *GP* general practitioner; *SCD* significant colorectal disease. ^1^ Non-SCD was established by other bowel tests for six patients (abdominal ultrasound in five and barium enema in one patient) and by the gastroenterologist based on bowel investigations performed before recruitment in the study for four patients. ^2^ SCD was established by the gastroenterologist for one patient on the basis of bowel investigations performed before recruitment in the study

Of 843 enrolled patients, 810 could be evaluated (96.1 %; Fig. 1). Their median age was 61 years (range 19–92), and 54.9 % were female. SCD was diagnosed in 17.4 % of patients (n = 141; 37 had CRC, 37 IBD, 18 diverticulitis, and 49 AA). The most frequent presenting symptoms were abdominal pain (80.7 %), change in bowel habit (65.5 %), constipation (57.9 %) and abdominal bloating (55.0 %; Table 1). CRP was elevated in 9.4 % and 48.7 % tested positive for calprotectin (POC, threshold at > 50 μg/g). Rectal blood loss was present in 43.6 % and 25.1 % tested POC FIT positive. Half the patients provided a faecal sample within 19 days of the GP visit (25^th^–75^th^ percentile: 13–26), median waiting time for endoscopy was 28 days (25^th^–75^th^ percentile: 17–39), and median time between faecal sample collection and endoscopy was 5 days (25^th^–75^th^ percentile: 1–15). Of all considered predictors, the faecal biomarkers yielded the highest NPVs for SCD if evaluated individually.

### Basic and extended diagnostic models

Nine of the 15 candidate predictors from patient history and physical examination were selected for the basic diagnostic model, to which blood Hb did not significantly contribute (*P* = 0.23) but CRP did (*P* = 0.03; see Table 2 for specification of the basic diagnostic model). This basic model significantly improved upon individual or combined extension with the calprotectin POC or ELISA and the POC FIT tests. Although CRP significantly contributed to the basic diagnostic model, it did not contribute to any of the five faecal biomarker extended models and was thus excluded from these. In none of the models with POC FIT did the odds ratio for SCD significantly differ in patients with and without rectal blood loss (Additional file 1), so we did not stratify the FIT results for overt rectal bleeding subgroups in the final models.

**Table 2:** Improvement in discrimination, reclassification, and explained variation upon various extensions of the basic diagnostic model and individual faecal biomarker extended models for SCD, as observed in 810 Dutch patients with lower abdominal complaints referred for endoscopy in the CEDAR study

	Absolute change in AUC		NRI^c^		IDI		Relative IDI, %	Absolute change in R^2^, %^d^
Diagnostic model	Estimate (95 % CI)	*P* value^b^	Estimate (95 % CI)	*P* value	Estimate (95 % CI)	*P* value		
Basic diagnostic model (AUC: 0.741; 95 % CI, 0.694–0.789)^a^								
+ Calprotectin POC test	0.022 (0.00–0.05)	0.078	0.15 (0.07–0.24)	<0.001	0.04 (0.02–0.06)	<0.001	27.6	4.5
+ Calprotectin ELISA test	0.019 (0.00–0.04)	0.11	0.13 (0.04–0.22)	0.005	0.04 (0.01–0.06)	0.005	27.5	4.3
+ POC FIT	0.090 (0.06–0.12)	<0.001	0.38 (0.24–0.51)	<0.001	0.13 (0.09–0.16)	<0.001	91.0	15.4
+ POC FIT and calprotectin POC test	0.096 (0.07–0.12)	<0.001	0.38 (0.25–0.51)	<0.001	0.14 (0.10–0.18)	<0.001	100.0	16.7
+ POC FIT and calprotectin ELISA test	0.096 (0.07–0.12)	<0.001	0.40 (0.27–0.52)	<0.001	0.14 (0.10–0.18)	<0.001	100.7	16.8
Calprotectin POC test extended model (AUC: 0.763; 95 % CI, 0.718–0.809)								
+ POC FIT	0.074 (0.05–0.10)	<0.001	0.23 (0.11–0.35)	<0.001	0.10 (0.07–0.13)	<0.001	56.9	12.3
Calprotectin ELISA test extended model (AUC: 0.760; 95 % CI, 0.714–0.806)								
+ POC FIT	0.077 (0.05–0.10)	<0.001	0.26 (0.14–0.39)	<0.001	0.10 (0.07–0.13)	<0.001	57.5	12.4
POC FIT extended model (AUC: 0.831; 95 % CI, 0.791–0.872)								
+ Calprotectin POC test	0.006 (0.00–0.02)	0.20	0.01 (–0.06 to 0.07)	0.87	0.01 (0.00–0.03)	0.055	4.7	1.3
+ Calprotectin ELISA test	0.005 (0.00–0.01)	0.20	0.02 (–0.05 to 0.09)	0.59	0.01 (0.00–0.03)	0.064	5.0	1.3

*AUC* area under the receiver operating characteristic curve; *CEDAR* Cost-Effectiveness of a Decision rule for Abdominal complaints in primary caRe; *CI* confidence interval; *CRP* C-reactive protein; *ELISA* enzyme-linked immunosorbent assay; *FIT* faecal immunochemical test for haemoglobin; *IDI* integrative discrimination improvement; *NRI* net reclassification improvement; *POC* point-of-care; *SCD* significant colorectal disease

^a^The basic diagnostic model consisted of: Age, per 5 years (OR: 1.2 (95 % CI, 1.1–1.3); *P* < 0.001); Abdominal pain (0.6 (0.4–0.9); *P* = 0.02); Rectal blood loss (3.1 (2.0–4.8); *P* < 0.001); Rectal mucus (1.8 (1.2–2.7); *P* = 0.007); Weight loss (1.5 (1.0–2.5); *P* = 0.08); Change in bowel habit (1.4 (0.8–2.2); *P* = 0.23); Abdominal bloating (0.6 (0.4–1.0); *P* = 0.04); Constipation (0.7 (0.5–1.1); *P* = 0.12); Abnormal digital rectal examination (1.7 (0.8–3.9); *P* = 0.20); CRP in mg/L, per log(CRP + 1) (1.3 (1.0–1.6); *P* = 0.03); CRP was included in the basic model, but omitted from the faecal biomarker extended models as it lost statistical significance (Additional file 1)

^b^ *P* value based on 2000-fold bootstrap resampling

^c^NRI is the NRI categorical with 5.0 % threshold for low and 50.0 % for high significant colorectal disease risk

^d^Nagelkerke’s R^2^

### Model performance and comparison

**Fig. 2:**
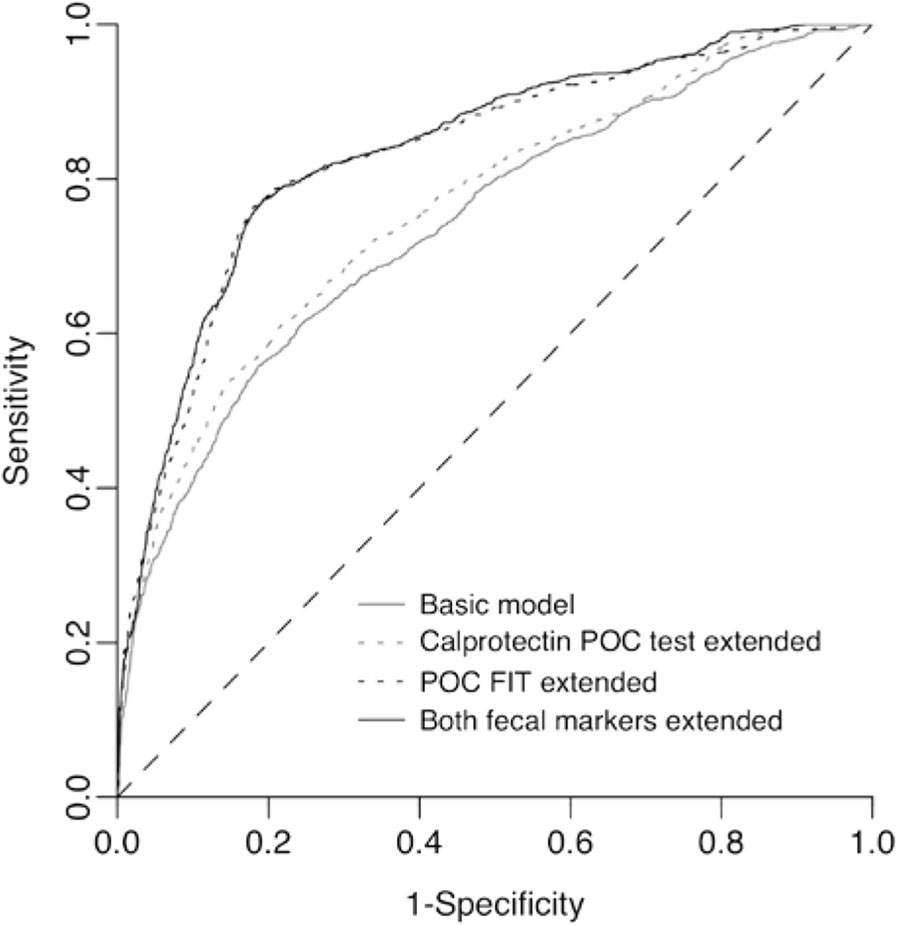
Receiver operating characteristic curves for diagnosing SCD for the basic diagnostic model, and the POC FIT and the calprotectin POC test extended models. *FIT* faecal immunochemical test for haemoglobin; *POC* point-of-care; *SCD* significant colorectal disease. Areas under the curve (before optimism-correction): basic model 0.741 (95 % CI, 0.694–0.789); calprotectin POC test extended 0.763 (95 % CI, 0.718–0.809); POC FIT extended 0.831 (95 % CI, 0.791–0.872); Both faecal POC tests extended 0.837 (95 % CI, 0.798–0.876). Dashed line is reference line

The basic model’s AUC increased from 0.741 (95 % CI, 0.694–0.789) to 0.763 (95 % CI, 0.718–0.809; *P* = 0.078) and 0.831 (95 % CI, 0.791–0.872; *P* < 0.001) upon extension with POC calprotectin and FIT, respectively, and to 0.837 (95 % CI, 0.798–0.876; *P* < 0.001) upon combined extension (Fig. 2 and Table 2). All three POC test extended models showed significant net reclassification improvement compared to the basic model. The FIT-only extended model and the combined POC extended model both yielded the highest NRI (both 0.38; see Additional file 1 for the corresponding reclassification tables). When adding FIT to the calprotectin POC extended model, both the AUC and NRI significantly increased, which was not true for adding calprotectin to the FIT extended model (Table 2). The basic model explained 19.0 % of the variation in SCD, which increased to 23.5, 34.5, and 35.8 % for the calprotectin, the FIT, and the combined POC extended models, respectively. All diagnostic models showed excellent calibration (Additional file 1).

### Ruling out SCD

Using the combined POC extended model at the ≥ 5.0 % SCD probability threshold for referral would rule out SCD (i.e. prevent referral) in 30.4 % of all patients in our study, with 96.4 % NPV and 93.7 % sensitivity (inappropriately not referring one CRC [stage 1], four diverticulitis, and four AA patients; Table 3). At the same threshold, the FIT-only extended model would rule out SCD in 30.1 % of patients with 96.0 % NPV, but would miss one additional AA (resulting in 93.0 % sensitivity). At a ≥ 2.5 % referral threshold, the considered diagnostic models would prevent referral in 2.0–7.2 % of patients with 98.0–100.0 % NPV and 99.4–100.0 % sensitivity, and a threshold of ≥ 7.5 % would prevent referral in 27.5–46.7 % of patients with 93.4–95.7 % NPV and 87.9–90.0 % sensitivity.

**Table 3:** Diagnostic accuracy when basing endoscopy referral on varying SCD probability thresholds for the basic and the five faecal biomarker extended models, as observed in 810 Dutch patients with lower abdominal complaints referred for endoscopy in the CEDAR study^a^

	Referred, % (95 % CI)	SCD detected, n	Missed SCD^b^, n					Accuracy for SCD			
Diagnostic model SCD probability threshold			Total	CRC	IBD	D	AA	Sensitivity, % (95 % CI)	PPV, % (95 % CI)	Specificity, % (95 % CI)	NPV, % (95 % CI)
Basic											
≥2.5 %	98.0 (96.4–99.2)	141	0	–	–	–	–	99.7 (96.7–100.0)	17.7 (15.2–20.5)	2.3 (1.0–4.1)	98.0 (76.5–100.0)
≥5.0 %	86.9 (83.9–89.6)	136	5	–	–	2	3	97.0 (92.6–98.8)	19.4 (16.6–22.5)	15.2 (12.1–18.8)	95.9 (90.2–98.5)
≥7.5 %	72.5 (69.1–75.6)	126	15	1	–	4	10	89.5 (83.2–93.8)	21.5 (18.3–25.0)	31.1 (27.5–35.0)	93.4 (89.2–96.1)
Calprotectin POC extended											
≥2.5 %	97.9 (96.4–98.9)	141	0	–	–	–	–	100.0 (97.3–100.0)	17.8 (15.3–20.6)	2.6 (1.4–4.3)	100.0 (81.1–100.0)
≥5.0 %	83.6 (80.3–86.6)	138	3	–	–	1	2	97.4 (93.1–99.2)	20.3 (17.4–23.5)	19.3 (15.8–23.3)	97.2 (92.6–99.2)
≥7.5 %	68.5 (64.9–71.9)	125	16	1	1	5	9	88.1 (81.5–92.7)	22.4 (19.1–26.1)	35.6 (31.7–39.7)	93.4 (89.6–96.0)
Calprotectin ELISA extended											
≥2.5 %	97.6 (96.0–98.8)	141	0	–	–	–	–	100.0 (97.3–100.0)	17.8 (15.3–20.7)	2.9 (1.5–4.8)	100.0 (82.7–100.0)
≥5.0 %	84.0 (81.1–86.6)	135	6	–	1	2	3	96.3 (91.5–98.7)	20.0 (17.1–23.2)	18.6 (15.5–22.0)	96.0 (90.7–98.6)
≥7.5 %	68.1 (64.7–71.4)	126	15	–	1	5	9	90.0 (83.7–94.2)	23.0 (19.7–26.7)	36.5 (32.8–40.4)	94.5 (91.0–96.8)
POC FIT extended											
≥2.5 %	92.8 (89.0–96.2)	140	1	–	–	–	1	99.4 (96.2–100.0)	18.6 (16.0–21.6)	8.6 (4.5–13.1)	98.7 (91.2–99.9)
≥5.0 %	69.9 (64.4–75.3)	131	10	1	–	4	5	93.0 (87.4–96.4)	23.2 (19.6–27.1)	34.9 (28.7–41.4)	96.0 (92.6–97.9)
≥7.5 %	54.3 (49.6–59.1)	124	17	1	4	6	6	88.1 (81.4–92.8)	28.2 (24.0–32.9)	52.8 (47.4–58.1)	95.5 (92.7–97.2)
Calprotectin POC and POC FIT extended model											
≥2.5 %	92.8 (89.4–95.7)	141	0	–	–	–	–	100.0 (97.3–100.0)	18.8 (16.1–21.8)	8.7 (5.2–12.8)	100.0 (93.4–100.0)
≥5.0 %	69.6 (64.7–74.3)	132	9	1	–	4	4	93.7 (88.2–96.8)	23.5 (19.9–27.3)	35.5 (29.9–41.2)	96.4 (93.1–98.2)
≥7.5 %	53.3 (48.7–57.8)	124	17	1	3	7	6	88.4 (81.7–93.1)	28.9 (24.7–33.5)	54.1 (49.1–59.1)	95.7 (93.1–97.4)
Calprotectin ELISA and POC FIT extended model											
≥2.5 %	92.8 (89.5–95.6)	141	0	–	–	–	–	100.0 (97.3–100.0)	18.8 (16.1–21.8)	8.7 (5.3–12.7)	100.0 (93.5–100.0)
≥5.0 %	69.0 (64.8–73.0)	132	9	1	–	4	4	93.3 (87.7–96.7)	23.6 (20.2–27.3)	36.2 (31.6–40.9)	96.3 (93.0–98.1)
≥7.5 %	53.4 (49.2–57.6)	124	17	1	2	7	7	87.9 (81.0–92.7)	28.7 (24.4–33.3)	53.9 (49.2–58.5)	95.5 (92.7–97.3)

*AA* advanced adenoma, *CEDAR* Cost-Effectiveness of a Decision rule for Abdominal complaints in primary caRe, *CI* confidence interval, *CRC* colorectal cancer, *D* diverticulitis, *ELISA* enzyme-linked immunosorbent assay, *FIT* faecal immunochemical test for haemoglobin, *IBD* inflammatory bowel disease, *NPV* negative predictive value, *POC* point-of-care, *PPV* positive predictive value, *SCD* significant colorectal disease

^a^The percentage referred and the accuracy measures are each averaged over the 10 imputed datasets. Hence, it is possible that, e.g. 100.0 % sensitivity does not directly match with 100.0 % NPV

^b^A patient with SCD was considered missed if his/her predicted SCD probability was below the respective threshold for referral in at least 5 of the 10 imputed datasets

Regarding the net benefit at the ≥ 5.0 % SCD probability threshold for referral when compared to the basic model, the combined POC extended model resulted in 60 more correctly non-referred patients without increasing the number of non-referred SCD patients, and three more correctly referred SCD patients without increasing unnecessary referrals (all per 1000 tested patients). These numbers were 34 and two, respectively, for the FIT extended model (Additional file 1).

### Calprotectin POC versus ELISA test

Substituting the calprotectin POC with an ELISA test yielded similar results with regard to discrimination, explained variation, reclassification, and diagnostic accuracy (Tables 2 and 3; see Additional file 1 for ROC curves).

### Towards use in new patients

To improve valid estimation of SCD risk in future patients, Table 4 shows the optimism-corrected regression coefficients of the combined POC and the FIT-only extended models (see Additional file 1 for the other models); the optimism-corrected AUC and explained variation of these models were 0.818 (95 % CI, 0.779–0.857) and 0.813 (95 % CI, 0.772–0.853), and 30.6 % and 29.5 %, respectively. See Additional file 1 for nomograms.

**Table 4:** Risk of SCD in relation to routine diagnostic predictors and faecal biomarkers as based on the optimism-corrected combined POC and the POC FIT extended diagnostic models, developed in 810 Dutch primary care patients with lower abdominal complaints referred for endoscopy in the CEDAR study^a,b^

	Calprotectin POC and POC FIT extended model			POC FIT extended model		
Diagnostic predictor	Regression coefficient (SE)	OR (95 % CI)	Wald *P* value	Regression coefficient (SE)	OR (95 % CI)	Wald *P* value
Patient history						
Age, per 5 years	0.11 (0.05)	1.1 (1.0–1.2)	0.024	0.12 (0.05)	1.1 (1.0–1.2)	0.008
Abdominal pain	–0.20 (0.27)	0.8 (0.5–1.4)	0.45	–0.22 (0.27)	0.8 (0.5–1.3)	0.4
Rectal blood loss	0.75 (0.25)	2.1 (1.3–3.4)	0.003	0.82 (0.25)	2.3 (1.4–3.7)	<0.001
Rectal mucus	0.37 (0.24)	1.4 (0.9–2.3)	0.13	0.43 (0.24)	1.5 (1.0–2.4)	0.072
Weight loss	0.27 (0.27)	1.3 (0.8–2.2)	0.33	0.34 (0.27)	1.4 (0.8–2.4)	0.21
Change in bowel habit	0.16 (0.28)	1.2 (0.7–2.0)	0.56	0.21 (0.28)	1.2 (0.7–2.1)	0.46
Abdominal bloating	–0.49 (0.24)	0.6 (0.4–1.0)	0.043	–0.49 (0.24)	0.6 (0.4–1.0)	0.044
Constipation	–0.23 (0.24)	0.8 (0.5–1.3)	0.33	–0.19 (0.24)	0.8 (0.5–1.3)	0.43
Physical examination						
Abnormal digital rectal examination	0.43 (0.49)	1.5 (0.6–4.0)	0.39	0.47 (0.47)	1.6 (0.6–4.0)	0.33
Faecal tests						
Calprotectin POC test, per 100 μg/g	0.28 (0.11)	1.3 (1.1–1.7)	0.014	–	–	–
Positive POC FIT (>6 μg Hb/g^c^)	1.75 (0.25)	5.8 (3.5–9.3)	<0.001	1.91 (0.24)	6.7 (4.2–10.7)	<0.001
Intercept	–4.08 (0.72)			–4.16 (0.72)		
AUC (95 % CI)	0.818 (0.779–0.857)			0.813 (0.772–0.853)		
Nagelkerke’s R^2^, % (95 % CI)	30.6 (22.4–39.0)			29.5 (21.2–37.9)		

*AUC* area under the receiver operating characteristic curve; *CEDAR* Cost-Effectiveness of a Decision rule for Abdominal complaints in primary caRe; *CI* confidence interval; *FIT* faecal immunochemical test for haemoglobin; *OR* odds ratio; *POC* point-of-care; *SCD* significant colorectal disease; *SE* standard error

^a^All regression coefficients, odds ratios, AUCs, and Nagelkerke’s R^2^s are optimism-corrected by 500-fold bootstrap resampling. Confidence intervals and Wald tests are based on optimism-corrected parameter estimates and assuming the same SE applies as before optimism-correction

^b^These models can be used to calculate the probability for a certain patient of having SCD. For example, according to the POC FIT extended model, a 60-year-old male patient with weight loss and a positive POC FIT has a 1/(1 + exp(–1 × (–4.16 + (0.12 × 60/5) – (0.22 × 0) + (0.82 × 0) + (0.43 × 0) + (0.34 × 1) + (0.21 × 0) – (0.49 × 0) – (0.19 × 0) + (0.47 × 0) + (1.91 × 1)))) = 38.5 % probability of having SCD. Similarly, according to the same model, a 60-year-old female patient with abdominal pain and bloating has a 3.1 % probability of having SCD

^c^Lower detection limit as stated by manufacturer

## Discussion

We are the first to develop a diagnostic strategy in primary care patients suspected of SCD, considering signs, symptoms, simple blood analyses, and both faecal calprotectin and Hb levels. This study showed that especially a POC FIT, and to a much lesser extent calprotectin tests, have incremental value beyond patient history, physical examination, and CRP in ruling out SCD in primary care patients with persistent lower abdominal complaints. Use of a simple diagnostic model including calprotectin POC and POC FIT test results could safely rule out SCD and prevent endoscopy referral in about 30 % of patients with 96.4 % NPV (at a 5.0 % SCD probability referral threshold). Excluding the calprotectin test from this model yielded similar results, missing one additional AA patient (of the 49 present in our study). Substituting the calprotectin POC test by an ELISA did not substantially change these results.

A perfect strategy would not miss any SCD patients. A substantial reduction of the number of unnecessary endoscopy referrals – as we show is feasible – will, however, inevitably result in a small risk of missing serious SCD. In our study, one patient with stage 1 CRC was not selected for referral by any of the POC FIT extended models at the ≥ 5.0 % SCD probability threshold (this patient tested negative on both the calprotectin POC test and the POC FIT). With keen attention in case of non-referral at first consultation to persisting symptoms over a time frame of 2–3 weeks, we think this will result in delaying, but not missing, such diagnoses. Such a limited delay will also not likely advance the disease stage substantially for CRC patients who were initially non-referred [29].

Notwithstanding the 2013 NICE recommendation for use in diagnosing IBD [10], calprotectin has so far only been studied in absence of other diagnostic information [11–13]. One retrospective study investigating the use of calprotectin in irritable bowel syndrome-suspected primary care patients from the United Kingdom reported an AUC for SCD of 0.89 (95 % CI, 0.85–0.93), much higher than we report here (0.68; 95 % CI, 0.63–0.73 [POC], 0.66; 95 % CI, 0.61–0.72 [ELISA]) [12]. Besides the different patient populations, adenomas were not considered SCD in that study, as they were in ours. As calprotectin levels are low in (advanced) adenoma patients [11], this partly explains the observed difference between the studies (AUCs for SCD without adenomas in our data: 0.74; 95 % CI, 0.69–0.80 [POC], 0.73; 95 % CI, 0.67–0.80 [ELISA]). Related to this, the prevalence of AAs in our study almost doubled from February 2011 onwards (from 4.2 to 7.7 %, comprising 25.8 % versus 41.8 % of SCD cases – an increase that could not be explained by changes in patient mix throughout the study period, nor by differences in detection rates between endoscopy centres, but may have been introduced by increased awareness of gastroenterologists who around that time started preparing for the introduction of the CRC screening program in 2014). This increase in AA prevalence likely explains why our current results are less favourable compared to our previous (interim) analysis of patients enrolled through January 2011 (AUC: 0.75; 95 % CI, 0.67–0.82 [POC], 0.73; 95 % CI, 0.66–0.81 [ELISA]) [11]. Still, calprotectin did not show as much incremental diagnostic value as expected. This observation remained when analysing the data for IBD instead of SCD, and when considering adenomas non-SCD (data not shown).

Faecal Hb testing for CRC screening is widely accepted. Here, we showed that a qualitative POC FIT also has large incremental value for ruling out SCD in primary care. Our data further suggests that the POC FIT has value even in patients with overt rectal bleeding, equally so as in those without (Additional file 1). Additional analysis showed that the POC FIT was negative in 65.6 % of our patients with overt rectal bleeding. It may be more specific for blood mixed with faeces, thereby better reflecting the generally higher gastrointestinal location of SCD compared to other causes of rectal bleeding (e.g. haemorrhoids).

In a recent United Kingdom-based primary care study that ran between 2013–2014, 755 patients referred for bowel examination had available data on both faecal calprotectin (same ELISA as in our study) as well as Hb levels (using the quantitative EIKEN OC-Sensor assay) [16]. The authors concluded that undetectable faecal Hb may be sufficient to exclude CRC/IBD/higher-risk adenomas with 41.7 % test negatives, 96.2 % NPV and 88.2 % sensitivity – thereby questioning the added value of calprotectin, as in our study. Other studies have also advocated quantitative faecal Hb testing for ruling out SCD [30, 31], or advanced neoplasia [32–34], in symptomatic patients. We could not confirm these promising results of faecal Hb by itself (Table 1), which is possibly because of the higher threshold of our POC FIT (with a detection limit of 6 μg/g), and it being a qualitative and not a quantitative test. Previous results suggest that using a single test could, in fact, be sufficient in deciding whom to refer for endoscopy. Indeed, our results also underscore that a positive POC FIT already implies the need for referral by itself (at the ≥ 5.0 % SCD probability threshold; see nomogram in Additional file 1). Here, the clinical data do not add much, but they do when the POC FIT returns negative. Also, in daily clinical practice, and certainly in primary care, it is rare that – except in a screening situation – physicians would immediately apply such test in suspected patients presenting with symptoms and signs of SCD without even considering any other pre-test diagnostic information from history taking and physical examination. The diagnostic process in primary care is sequential, starting with history taking and physical examination, and follow-up testing only in cases where the first provide indications that legitimates additional testing. To adhere as much as possible to primary care practice, we therefore explicitly first evaluated the diagnostic value of history taking, physical examination, and simple blood analysis, and subsequently the added value of the POC FIT test, rather than the other way around. Obviously, in unsuspected people, in the realm of screening, a single-test approach using first and foremost the POC FIT test, seems a very reasonable approach, but in our view not for diagnostic work-up of clinically suspected patients, which was the focus of this paper.

A major strength of our study is its prospective conduct in a primary care setting, where results from secondary care studies may not be applicable [8]. We also took care to enrol representative patients from 266 general practices, while measuring all potentially relevant diagnostic information, including blood and faecal biomarkers, under routine conditions, enhancing the generalizability of our results. Moreover, patients underwent reference testing by the same standard, including 3 months follow-up after inconclusive endoscopy to identify any initially missed SCD, and index and reference tests were interpreted independently in each patient. Finally, we purposely developed diagnostic models for SCD, and not solely for CRC (or IBD) as commonly done. This resulted in a diagnostic strategy applicable to primary care patients with persistent lower abdominal complaints that is optimally aligned with the diagnostic challenge at hand: ruling out SCD.

When defining SCD, we only included adenomas > 1 cm as AA, without taking histologic high-risk features such as the presence of high-grade dysplasia or villous components in smaller adenomas into account. However, such high-risk features are seldom present in small adenomas [35], and we estimate that about 2 to 3 of the small adenomas we have considered non-SCD are actually high-risk lesions. This amount of misclassification (i.e. only ~2 % of all SCD cases in CEDAR) will likely not have importantly influenced the results. Some other limitations of our study also need discussion. For instance, we did not enrol primary care patients urgently referred for endoscopy (e.g. for on-going bleeding or imminent obstruction) or at very low SCD-suspicion (not necessitating endoscopy). Our study population thus reflects patients at intermediate risk of SCD. These patients, however, pose the largest diagnostic dilemma, where an improved diagnostic work-up is especially urgent. Further, most diagnostic predictors had missing data despite systematic data collection, and we had to use state of the art multiple imputation of the 5.2 % missing data points to prevent selection bias and loss of information [23–25]. Furthermore, as we used all available data to optimally develop the best diagnostic strategy, and despite using bootstrapping techniques for internal validation to correct for over-optimism, formal external validation of our findings is still warranted.

Finally, the use of a qualitative POC FIT in the way that we did in this study, although easily implemented in primary care, also has limitations. First, as the qualitative POC FIT yields a positive or a negative test result (with a detection limit of 6 μg Hb/g faeces), the diagnostic information that would be available by quantitatively assessing the amount of Hb present in faeces is lost. Second, patients collected faecal samples in regular blue-capped containers without Hb stabilizing buffer (so each patient needed to fill only one faecal container for both calprotectin and Hb analysis). Samples were kept refrigerated, and – if not frozen before further processing – 90 % were tested within 3 days of collection. Additional data-analysis showed that the chance of a positive POC FIT slightly decreased with increasing time between collection and testing (0.3 % absolute decrease per day; *P* = 0.19), and that frozen samples were more likely to be POC FIT negative than non-frozen samples (absolute 8.6 % decrease in POC FIT positivity; *P* = 0.017; calprotectin results seemed not to be affected). Some patients have thus likely tested falsely negative for the POC FIT because of Hb degradation in our study. However, in none of the models with POC FIT did its odds ratio for SCD significantly differ in patients whose faecal samples were and were not frozen. Furthermore, the POC FIT performed well in our study despite these limitations, and the sensitivity and discriminatory performance of faecal Hb testing in primary care will thus likely be even better when using Hb stabilizing buffers in faecal sample collection devices and using a quantitative FIT.

## Conclusions

A simple model including information from history taking, physical examination, and a POC FIT may safely rule out SCD and prevent unnecessary endoscopy referral in approximately one-third of SCD-suspected primary care patients. Adding a calprotectin test to such a strategy has limited value.

## Additional file

Additional file 1: Supplementary appendix. Contents: interaction between POC FIT and overt rectal bleeding; **Table S1:** model development strategy and specification; **Table S2:** reclassification table combined POC extended model versus basic diagnostic model; **Table S3:** reclassification table POC FIT extended model versus basic diagnostic model; **Table S4:** optimism corrected parameters for the basic model, the calprotectin POC extended model, the calprotectin ELISA extended model, and the calprotectin ELISA and POC FIT extended model; **Figure S1:** calibration curves; **Figure S2:** decision curve analysis; **Figure S3:** ROC curves subsitituting calprotectin POC with ELISA; **Figure S4:** nomogram of the combined POC extended model; **Figure S5:** nomogram of the POC FIT extended model. (DOCX 2031 kb)

## Abbreviations

AA: advanced adenoma
AIC: Akaike’s information criterion
AUC: area under the receiver operating characteristic curve
CEDAR: Cost-Effectiveness of a Decision rule for Abdominal complaints in primary caRe
CRC: colorectal cancer
CRP: C-reactive protein
ELISA: enzyme-linked immunosorbent assay
FIT: faecal immunochemical test for haemoglobin
GP: general practitioner
Hb: haemoglobin
IBD: inflammatory bowel disease
IDI: integrated discrimination improvement
NICE: National Institute for Health and Care Excellence
NPV: negative predictive value
NRI: net reclassification improvement
POC: point-of-care
PPV: positive predictive value
ROC curve: receiver operating characteristic curve
SCD: significant colorectal disease
