# Supplementary material for: Is there an added value of faecal calprotectin and haemoglobin in the diagnostic work-up for primary care patients suspected of significant colorectal disease? A cross-sectional diagnostic study
Source: BMC Med. 2016 Sep 26;14:141. doi: 10.1186/s12916-016-0684-5 (PMC5036273; doi:10.1186/s12916-016-0684-5)
Supplement: Additional file 1: — Supplementary appendix. Contents: interaction between POC FIT and overt rectal bleeding; Table S1: model development strategy and specification; Table S2: reclassification table combined POC extended model versus basic diagnostic model; Table S3: reclassification table POC FIT extended model versus basic diagnostic model; Table S4: optimism corrected parameters for the basic model, the calprotectin POC extended model, the calprotectin ELISA extended model, and the calprotectin ELISA and POC FIT extended model; Figure S1: calibration curves; Figure S2: decision curve analysis; Figure S3: ROC curves subsitituting calprotectin POC with ELISA; Figure S4: nomogram of the combined POC extended model; Figure S5: nomogram of the POC FIT extended model. (DOCX 2031 kb) [file 12916_2016_684_MOESM1_ESM.docx]

# Supplementary appendix

Supplement to: Elias SG, Kok L, de Wit NJ et al. Is there an added value of faecal calprotectin and haemoglobin in the diagnostic work-up for primary care patients suspected of significant colorectal disease? A cross-sectional diagnostic study

**Table of Contents**

Interaction between POC FIT and overt rectal bleeding 3

Table S1. Model development strategy and specification 4

Table S2. Reclassification table combined POC extended model versus basic diagnostic model 5

Table S3. Reclassification table POC FIT extended model versus basic diagnostic model 6

Table S4. Optimism corrected model parameters for the basic model, the calprotectin POC extended model, the calprotectin ELISA extended model, and the calprotectin ELISA and POC FIT extended model 7

Figure S1. Calibration curves 8

Figure S2. Decision curve analysis 9

Figure S3. ROC curves substituting calprotectin POC with ELISA 10

Figure S4. Nomogram of the combined POC extended model 11

Figure S5. Nomogram of the POC FIT extended model 12

**Interaction between POC FIT and overt rectal bleeding**

Of all patients, 46.3% reported no rectal bleeding and tested negative on the POC FIT, 10.0% reported no rectal bleeding but tested positive on the POC FIT, 28.5% reported rectal bleeding but tested negative on the POC FIT, and 15.1% reported rectal bleeding and tested positive on the POC FIT.

To evaluate whether a POC FIT still has value for diagnosing significant colorectal disease in patients who report to have overt rectal bleeding, we evaluated and tested an interaction term [FIT*blood loss] in the three models containing the POC FIT test (the POC FIT extended model, the combined POC extended model, and the POC FIT and calprotectin ELISA extended model):

**Odds ratio (95%CI) for SCD**

**of POC FIT positive versus negative result**

**In POC FIT extended model**

Overall (no interaction): 8.1 (5.1-12.8)

If no rectal bleeding: 7.0 (3.6-13.9)

If rectal bleeding: 8.9 (4.8-16.3)

*p-interaction [FIT*blood loss]: 0*.*62*

**In combined POC extended model**

Overall (no interaction): 6.8 (4.2-11.1)

If no rectal bleeding: 6.2 (3.1-12.3)

If rectal bleeding: 7.4 (3.9-13.9)

*p-interaction [FIT*blood loss]: 0*.*70*

**In POC FIT and calprotectin ELISA extended model**

Overall (no interaction): 6.9 (4.3-11.0)

If no rectal bleeding: 6.2 (3.1-12.4)

If rectal bleeding: 7.4 (4.0-13.8)

*p-interaction [FIT*blood loss]: 0*.*71*

Stratum-specific odds ratios are derived from a model with [FIT*blood loss] interaction tem. Above results are apparent results, i.e. not optimism-corrected.

| **Table S1.** Model development strategy and specification, and frequency of selection of individual predictors at each development step over 500 bootstrap-resamples. | | | |
| --- | --- | --- | --- |
|  | **Data-analysis** | | **Bootstrap-resampling (500)** |
| **Selection strategy and diagnostic predictors considered at each step** | Selection criterion and result | Consequence for predictor | Frequency of selection |
| **1. Development of the basic model** |  |  |  |
| Step 1a: Patient history |  |  |  |
| *Backward AIC-based selection of patient history predictors per multiple imputation dataset, retaining predictors if selected in at least half of the multiple imputation datasets* | No. out of 10 MI datasets  retaining the predictor |  |  |
| - Gender | 0 | Removed | 22.4% |
| - Age^1^ | 10 | **Selected** | 99.8% |
| - Abdominal pain | 10 | **Selected** | 88.8% |
| - Duration of abdominal pain^1^ | 0 | Removed | 21.4% |
| - Rectal blood loss | 10 | **Selected** | 100.0% |
| - Rectal mucus | 10 | **Selected** | 96.0% |
| - Weight loss | 10 | **Selected** | 56.6% |
| - Change in bowel habit | 6 | **Selected** | 57.2% |
| - Abdominal bloating | 10 | **Selected** | 83.2% |
| - Fever | 0 | Removed | 19.0% |
| - Constipation | 7 | **Selected** | 48.8% |
| - Diarrheal | 0 | Removed | 26.2% |
| - Family history of colorectal cancer | 0 | Removed | 20.4% |
|  |  |  |  |
| Step 1b: Physical examination |  |  |  |
| *Addition of physical examination predictors to selected patient history predictors and subsequent backward AIC-based selection as in Step 1a, fixing patient history selected predictors* | No. out of 10 MI datasets  retaining the predictor |  |  |
| - Palpable abdominal mass | 0 | Removed | 19.4% |
| - Digital rectal examination | 7 | **Selected** | 58.6% |
|  |  |  |  |
| Step 1c: Blood analyses |  |  |  |
| *Addition of blood analyses to selected patient history and physical examination predictors and forward likelihood ratio test-based selection at p<0.05* | LR p value |  |  |
| - Haemoglobin^1^ | 0.23 | Not selected | 22.4% |
| - C-reactive protein^1^ | 0.03 | **Selected** | 55.2% |
|  |  |  |  |
| **2. Development of the faecal biomarker extended models** |  |  |  |
| *Addition of individual faecal biomarker tests as well as calprotectin and FIT combinations to the basic model (5 models). Forward likelihood ratio test-based selection at p<0.05 versus the basic model for single marker extensions, and versus each single marker extended model for the combination extensions (both then need to show p<0.05)* | LR p value |  |  |
| - Calprotectin POC test^1^ | <0.001 | **Selected** | 98.6% |
| - Calprotectin ELISA test^1^ | <0.001 | **Selected** | 99.2% |
| - POC FIT | <0.001 | **Selected** | 99.0% |
| - POC FIT and calprotectin POC test^1^ | <0.001; 0.01^2^ | **Selected** | 65.0% |
| - POC FIT and calprotectin ELISA test^1^ | <0.001; 0.02^2^ | **Selected** | 64.0% |
|  |  |  |  |
| **3. Specification of the final models: evaluate redundancy of blood analyses** |  |  |  |
| *If blood analysis is present in the faecal biomarker extended models, backward likelihood ratio test-based selection at p≥0.05 for removal* | LR p value |  |  |
| - Haemoglobin^1^ | NA | NA | 16.4% |
| - C-reactive protein^1^ | all > 0.28 | Removed | 6.1% |

Abbreviations: AIC: Akaike information criterion; ELISA: enzyme-linked immunosorbent assay; FIT: faecal immunochemical test for haemoglobin; LR: likelihood ratio; MI: multiple imputation; NA: not applicable; POC: point-of-care.

^1^ Coding of continuous predictors: 1) age: linear; 2) duration of abdominal pain: logarithmic, truncated at 10 years and centred around 90 days for those with abdominal pain; 3) haemoglobin: linear and quadratic; 4) C-reactive protein: logarithmic+1, truncated at 20 mg/L; 5) calprotectin POC: linear; 6) calprotectin ELISA: linear, truncated at 500 μg/g;

^2^ First p value for addition of POC FIT to calprotectin extended model, second p value for addition of calprotectin to POC FIT extended model;

^3^ These combined biomarker models were not selected as often, predominantly as the addition of the calprotectin test to a POC FIT extended model did not improve the model significantly, but compared to the basic model these combined faecal biomarker models showed highly significant better fits.

| **Table S2.** Reclassification of SCD risk when comparing a model with routine diagnostic predictors (basic model) with the calprotectin POC test and POC FIT extended model, as observed in 810 Dutch patients with lower abdominal complaints referred for endoscopy in the CEDAR study.^1^ | | | | | | | | | | | | | | | | | | |
| --- | --- | --- | --- | --- | --- | --- | --- | --- | --- | --- | --- | --- | --- | --- | --- | --- | --- | --- |
| **A. Overall reclassification and actual SCD risk across risk categories** | | | | | | | | | | | | | | | | | | |
|  |  |  |  |  |  |  |  |  |  |  |  |  |  |  |  |  |  |  |
|  | **Overall reclassification according to predicted risk categories** | | | | | | | | | |  |  |  |  |  |  |  |  |
|  |  |  |  |  |  |  |  |  |  |  |  |  |  |  |  |  |  |  |
|  |  |  |  | Calprotectin POC test and POC FIT extended model | | | | | | |  |  |  |  |  | **Movement across risk categories, n (%)** | | |
|  |  |  |  | <5% |  |  | 5-50% |  |  | ≥50% |  |  | **Total (%)** |  |  | Down | No change | Up |
|  |  | <5% |  | 91 | -> |  | 15 |  |  | - |  |  | 106 (13.1) |  |  | 165 (20.4) | 561 (69.3) | 84 (10.4) |
|  | Basic model | 5-50% |  | 155 |  | <- | 452 | -> |  | 69 |  |  | 677 (83.6) |  |  |  |  |  |
|  |  | ≥50% |  | - |  |  | 10 |  | <- | 18 |  |  | 27 (3.3) |  |  |  |  |  |
|  |  |  |  |  |  |  |  |  |  |  |  |  |  |  |  |  |  |  |
|  |  | **Total (%)** |  | 246 (30.4) |  |  | 477 (58.9) |  |  | 86 (10.6) |  |  | 810 |  |  |  |  |  |
|  |  |  |  |  |  |  |  |  |  |  |  |  |  |  |  |  |  |  |
|  |  |  |  |  |  |  |  |  |  |  |  |  |  |  |  |  |  |  |
|  | **Actual risk of SCD (95% CI) according to predicted risk categories** | | | | | | | | | |  |  |  |  |  |  |  |  |
|  |  |  |  |  |  |  |  |  |  |  |  |  |  |  |  |  |  |  |
|  |  |  |  | Calprotectin POC test and POC FIT extended model | | | | | | |  |  |  |  |  |  |  |  |
|  |  |  |  | <5% |  |  | 5-50% |  |  | ≥50% |  |  | **Total** |  |  |  |  |  |
|  |  | <5% | 2.5 (0.4-8.5) | | | 13.3 (1.6-40.3) | | | - | | | 4.1 (1.5-9.8) | | |  |  |  |  |
|  | Basic model | 5-50% | 4.3 (1.5-9.4) | | | 15.6 (12.3-19.4) | | | 59.0 (45.8-71.2) | | | 17.4 (14.7-20.4) | | |  |  |  |  |
|  |  | ≥50% | - | | | 59.2 (24.8-88.4) | | | 75.9 (50.8-91.7) | | | 70.0 (50.0-85.1) | | |  |  |  |  |
|  |  |  |  |  |  |  |  |  |  |  |  |  |  |  |  |  |  |  |
|  |  | **Total** | 3.6 (1.8-6.9) | | | 16.4 (13.2-20.1) | | | 62.5 (50.9-72.9) | | |  |  |  |  |  |  |  |
|  |  |  |  |  |  |  |  |  |  |  |  |  |  |  |  |  |  |  |
|  |  |  |  |  |  |  |  |  |  |  |  |  |  |  |  |  |  |  |
| **B. Reclassification according to SCD status** | | | | | | | | | | | | | | | | | | |
|  |  |  |  |  |  |  |  |  |  |  |  |  |  |  |  |  |  |  |
|  | **Number of patients with SCD according to predicted risk categories** | | | | | | | | | |  |  |  |  |  | **Net reclassification improvement (NRI) indices** | | |
|  |  |  |  |  |  |  |  |  |  |  |  |  |  |  |  |  |  |  |
|  |  |  |  | Calprotectin POC test and POC FIT extended model | | | | | | |  |  |  |  |  |  |  |  |
|  |  |  |  | <5% |  |  | 5-50% |  |  | ≥50% |  |  |  |  |  | **With SCD (95% CI) p value** | | |
|  |  | <5% |  | 2 | -> |  | 2 |  |  | - |  |  |  |  |  | NRI categorical | 0.22 (0.11-0.32) p<0.001 | |
|  | Basic model | 5-50% |  | 7 |  | <- | 70 | -> |  | 41 |  |  |  |  |  | NRI clinical | 0.29 (0.18-0.40) p<0.001 | |
|  |  | ≥50% |  | - |  |  | 6 |  | <- | 13 |  |  |  |  |  | NRI continuous | 0.37 (0.21-0.53) p<0.001 | |
|  |  |  |  |  |  |  |  |  |  |  |  |  |  |  |  |  |  |  |
|  |  |  |  |  |  |  |  |  |  |  |  |  |  |  |  |  |  |  |
|  |  |  |  |  |  |  |  |  |  |  |  |  |  |  |  |  |  |  |
|  | **Number of patients without SCD according to predicted risk categories** | | | | | | | | | |  |  |  |  |  |  |  |  |
|  |  |  |  |  |  |  |  |  |  |  |  |  |  |  |  |  |  |  |
|  |  |  |  | Calprotectin POC test and POC FIT extended model | | | | | | |  |  |  |  |  |  |  |  |
|  |  |  |  | <5% |  |  | 5-50% |  |  | ≥50% |  |  |  |  |  | **Without SCD (95% CI) p value** | | |
|  |  | <5% |  | 89 | -> |  | 13 |  |  | - |  |  |  |  |  | NRI categorical | 0.17 (0.11-0.23) p<0.001 | |
|  | Basic model | 5-50% |  | 149 |  | <- | 382 | -> |  | 28 |  |  |  |  |  | NRI clinical | 0.22 (0.15-0.28) p<0.001 | |
|  |  | ≥50% |  | - |  |  | 4 |  | <- | 4 |  |  |  |  |  | NRI continuous | 0.62 (0.54-0.69) p<0.001 | |
|  |  |  |  |  |  |  |  |  |  |  |  |  |  |  |  |  |  |  |
|  |  |  |  |  |  |  |  |  |  |  |  |  |  |  |  |  |  |  |
|  |  |  |  |  |  |  |  |  |  |  |  |  |  |  |  |  |  |  |
|  |  |  |  |  |  |  |  |  |  |  |  |  |  |  |  | **Overall (95% CI) p value** | | |
|  |  |  |  |  |  |  |  |  |  |  |  |  |  |  |  | NRI categorical | 0.38 (0.25-0.51) p<0.001 | |
|  |  |  |  |  |  |  |  |  |  |  |  |  |  |  |  | NRI clinical | 0.51 (0.36-0.65) p<0.001 | |
|  |  |  |  |  |  |  |  |  |  |  |  |  |  |  |  | NRI continuous | 0.99 (0.81-1.16) p<0.001 | |
|  |  |  |  |  |  |  |  |  |  |  |  |  |  |  |  |  |  |  |

Abbreviations: CEDAR: Cost-Effectiveness of a Decision rule for Abdominal complaints in primary caRe; CI: confidence interval; FIT: faecal immunochemical test for haemoglobin; NRI: net reclassification improvement; POC: point-of-care; SCD: significant colorectal disease.

^1^ Each result in this table is averaged over the 10 imputed datasets. Hence, it is possible that numbers do not match, e.g., adding the numbers from the reclassification tables in part B may not exactly match the numbers in the reclassification table in part A.

| **Table S3.** Reclassification of SCD risk when comparing a model with routine diagnostic predictors (basic model) with the POC FIT extended model, as observed in 810 Dutch patients with lower abdominal complaints referred for endoscopy in the CEDAR study.^1^ | | | | | | | | | | | | | | | | | | |
| --- | --- | --- | --- | --- | --- | --- | --- | --- | --- | --- | --- | --- | --- | --- | --- | --- | --- | --- |
| **A. Overall reclassification and actual SCD risk across risk categories** | | | | | | | | | | | | | | | | | | |
|  |  |  |  |  |  |  |  |  |  |  |  |  |  |  |  |  |  |  |
|  | **Overall reclassification according to predicted risk categories** | | | | | | | | | |  |  |  |  |  |  |  |  |
|  |  |  |  |  |  |  |  |  |  |  |  |  |  |  |  |  |  |  |
|  |  |  |  | POC FIT extended model | | | | | | |  |  |  |  |  | **Movement across risk categories, n (%)** | | |
|  |  |  |  | <5% |  |  | 5-50% |  |  | ≥50% |  |  | **Total (%)** |  |  | Down | No change | Up |
|  |  | <5% |  | 94 | -> |  | 12 |  |  | - |  |  | 106 (13.1) |  |  | 159 (19.6) | 562 (69.4) | 89 (11.0) |
|  | Basic model | 5-50% |  | 150 |  | <- | 450 | -> |  | 77 |  |  | 677 (83.6) |  |  |  |  |  |
|  |  | ≥50% |  | - |  |  | 10 |  | <- | 18 |  |  | 27 (3.3) |  |  |  |  |  |
|  |  |  |  |  |  |  |  |  |  |  |  |  |  |  |  |  |  |  |
|  |  | **Total (%)** |  | 244 (30.1) |  |  | 472 (58.3) |  |  | 95 (11.7) |  |  | 810 |  |  |  |  |  |
|  |  |  |  |  |  |  |  |  |  |  |  |  |  |  |  |  |  |  |
|  |  |  |  |  |  |  |  |  |  |  |  |  |  |  |  |  |  |  |
|  | **Actual risk of SCD (95% CI) according to predicted risk categories** | | | | | | | | | |  |  |  |  |  |  |  |  |
|  |  |  |  |  |  |  |  |  |  |  |  |  |  |  |  |  |  |  |
|  |  |  |  | POC FIT extended model | | | | | | |  |  |  |  |  |  |  |  |
|  |  |  |  | <5% |  |  | 5-50% |  |  | ≥50% |  |  | **Total** |  |  |  |  |  |
|  |  | <5% | 2.8 (0.7-8.5) | | | 13.9 (2.4-43.1) | | | - | | | 4.1 (1.5-9.8) | | |  |  |  |  |
|  | Basic model | 5-50% | 4.9 (2.1-9.9) | | | 15.0 (11.8-18.8) | | | 55.6 (43.9-66.7) | | | 17.4 (14.7-20.4) | | |  |  |  |  |
|  |  | ≥50% | - | | | 58.7 (23.8-88.7) | | | 76.1 (51.0-91.8) | | | 70.0 (50.0-85.1) | | |  |  |  |  |
|  |  |  |  |  |  |  |  |  |  |  |  |  |  |  |  |  |  |  |
|  |  | **Total** | 4.0 (2.1-7.4) | | | 15.9 (12.8-19.5) | | | 59.4 (48.8-69.3) | | |  |  |  |  |  |  |  |
|  |  |  |  |  |  |  |  |  |  |  |  |  |  |  |  |  |  |  |
|  |  |  |  |  |  |  |  |  |  |  |  |  |  |  |  |  |  |  |
| **B. Reclassification according to SCD status** | | | | | | | | | | | | | | | | | | |
|  |  |  |  |  |  |  |  |  |  |  |  |  |  |  |  |  |  |  |
|  | **Number of patients with SCD according to predicted risk categories** | | | | | | | | | |  |  |  |  |  | **Net reclassification improvement (NRI) indices** | | |
|  |  |  |  |  |  |  |  |  |  |  |  |  |  |  |  |  |  |  |
|  |  |  |  | POC FIT extended model | | | | | | |  |  |  |  |  |  |  |  |
|  |  |  |  | <5% |  |  | 5-50% |  |  | ≥50% |  |  |  |  |  | **With SCD (95% CI) p value** | | |
|  |  | <5% |  | 3 | -> |  | 2 |  |  | - |  |  |  |  |  | NRI categorical | 0.22 (0.12-0.33) p<0.001 | |
|  | Basic model | 5-50% |  | 7 |  | <- | 68 | -> |  | 43 |  |  |  |  |  | NRI clinical | 0.30 (0.19-0.42) p<0.001 | |
|  |  | ≥50% |  | - |  |  | 6 |  | <- | 13 |  |  |  |  |  | NRI continuous | 0.34 (0.18-0.50) p<0.001 | |
|  |  |  |  |  |  |  |  |  |  |  |  |  |  |  |  |  |  |  |
|  |  |  |  |  |  |  |  |  |  |  |  |  |  |  |  |  |  |  |
|  |  |  |  |  |  |  |  |  |  |  |  |  |  |  |  |  |  |  |
|  | **Number of patients without SCD according to predicted risk categories** | | | | | | | | | |  |  |  |  |  |  |  |  |
|  |  |  |  |  |  |  |  |  |  |  |  |  |  |  |  |  |  |  |
|  |  |  |  | POC FIT extended model | | | | | | |  |  |  |  |  |  |  |  |
|  |  |  |  | <5% |  |  | 5-50% |  |  | ≥50% |  |  |  |  |  | **Without SCD (95% CI) p value** | | |
|  |  | <5% |  | 91 | -> |  | 10 |  |  | - |  |  |  |  |  | NRI categorical | 0.15 (0.09-0.22) p<0.001 | |
|  | Basic model | 5-50% |  | 142 |  | <- | 382 | -> |  | 34 |  |  |  |  |  | NRI clinical | 0.19 (0.11-0.27) p<0.001 | |
|  |  | ≥50% |  | - |  |  | 4 |  | <- | 4 |  |  |  |  |  | NRI continuous | 0.67 (0.61-0.74) p<0.001 | |
|  |  |  |  |  |  |  |  |  |  |  |  |  |  |  |  |  |  |  |
|  |  |  |  |  |  |  |  |  |  |  |  |  |  |  |  |  |  |  |
|  |  |  |  |  |  |  |  |  |  |  |  |  |  |  |  |  |  |  |
|  |  |  |  |  |  |  |  |  |  |  |  |  |  |  |  | **Overall (95% CI) p value** | | |
|  |  |  |  |  |  |  |  |  |  |  |  |  |  |  |  | NRI categorical | 0.38 (0.24-0.51) p<0.001 | |
|  |  |  |  |  |  |  |  |  |  |  |  |  |  |  |  | NRI clinical | 0.50 (0.35-0.64) p<0.001 | |
|  |  |  |  |  |  |  |  |  |  |  |  |  |  |  |  | NRI continuous | 1.02 (0.85-1.19) p<0.001 | |
|  |  |  |  |  |  |  |  |  |  |  |  |  |  |  |  |  |  |  |

Abbreviations: CEDAR: Cost-Effectiveness of a Decision rule for Abdominal complaints in primary caRe; CI: confidence interval; FIT: faecal immunochemical test for haemoglobin; NRI: net reclassification improvement; POC: point-of-care; SCD: significant colorectal disease.

^1^ Each result in this table is averaged over the 10 imputed datasets. Hence, it is possible that numbers do not match, e.g., adding the numbers from the reclassification tables in part B may not match the numbers in the reclassification table in part A.

| **Table S4.** Risk of SCD in relation to routine diagnostic predictors and faecal biomarkers as based on the optimism-corrected models, developed in 810 Dutch primary care patients with lower abdominal complaints referred for endoscopy in the CEDAR study.^1^ | | | | | | | | | | | | |
| --- | --- | --- | --- | --- | --- | --- | --- | --- | --- | --- | --- | --- |
|  | **Routine diagnostic predictors (basic model)** | | | **Calprotectin POC extended model** | | | **Calprotectin ELISA extended model** | | | **Calprotectin ELISA and POC FIT extended model** | | |
| **Diagnostic predictor** | Regression coefficient (SE) | OR (95% CI) | Wald p value | Regression coefficient (SE) | OR (95% CI) | Wald p value | Regression coefficient (SE) | OR (95% CI) | Wald p value | Regression coefficient (SE) | OR (95% CI) | Wald p value |
| **Patient history** |  |  |  |  |  |  |  |  |  |  |  |  |
| Age, per 5 years | 0.14 (0.04) | 1.1 (1.1-1.2) | 0.002 | 0.11 (0.04) | 1.1 (1.0-1.2) | 0.01 | 0.13 (0.04) | 1.1 (1.0-1.2) | 0.004 | 0.11 (0.05) | 1.1 (1.0-1.2) | 0.016 |
| Abdominal pain | -0.47 (0.24) | 0.6 (0.4-1.0) | 0.047 | -0.46 (0.24) | 0.6 (0.4-1.0) | 0.057 | -0.51 (0.24) | 0.6 (0.4-1.0) | 0.039 | -0.24 (0.27) | 0.8 (0.5-1.3) | 0.38 |
| Rectal blood loss | 0.96 (0.22) | 2.6 (1.7-4.0) | < 0.001 | 0.87 (0.23) | 2.4 (1.5-3.7) | < 0.001 | 0.91 (0.23) | 2.5 (1.6-3.9) | < 0.001 | 0.76 (0.25) | 2.1 (1.3-3.5) | 0.002 |
| Rectal mucus | 0.50 (0.22) | 1.6 (1.1-2.5) | 0.022 | 0.44 (0.22) | 1.6 (1.0-2.4) | 0.049 | 0.42 (0.22) | 1.5 (1.0-2.4) | 0.063 | 0.36 (0.24) | 1.4 (0.9-2.3) | 0.14 |
| Weight loss | 0.37 (0.24) | 1.4 (0.9-2.3) | 0.13 | 0.27 (0.25) | 1.3 (0.8-2.1) | 0.28 | 0.25 (0.25) | 1.3 (0.8-2.1) | 0.32 | 0.26 (0.27) | 1.3 (0.8-2.2) | 0.35 |
| Change in bowel habit | 0.26 (0.26) | 1.3 (0.8-2.1) | 0.31 | 0.25 (0.26) | 1.3 (0.8-2.1) | 0.33 | 0.22 (0.26) | 1.2 (0.8-2.1) | 0.39 | 0.15 (0.28) | 1.2 (0.7-2.0) | 0.6 |
| Abdominal bloating | -0.40 (0.23) | 0.7 (0.4-1.0) | 0.076 | -0.44 (0.23) | 0.6 (0.4-1.0) | 0.057 | -0.39 (0.23) | 0.7 (0.4-1.1) | 0.091 | -0.46 (0.24) | 0.6 (0.4-1.0) | 0.059 |
| Constipation | -0.28 (0.22) | 0.8 (0.5-1.1) | 0.19 | -0.32 (0.22) | 0.7 (0.5-1.1) | 0.14 | -0.28 (0.22) | 0.8 (0.5-1.2) | 0.19 | -0.21 (0.24) | 0.8 (0.5-1.3) | 0.38 |
| **Physical examination** |  |  |  |  |  |  |  |  |  |  |  |  |
| Abnormal digital rectal examination | 0.46 (0.42) | 1.6 (0.7-3.6) | 0.28 | 0.41 (0.45) | 1.5 (0.6-3.6) | 0.36 | 0.47 (0.46) | 1.6 (0.7-3.9) | 0.31 | 0.46 (0.50) | 1.6 (0.6-4.2) | 0.36 |
| **Blood analyses** |  |  |  |  |  |  |  |  |  |  |  |  |
| C-reactive protein in mg/L, per log(CRP+1) | 0.21 (0.11) | 1.2 (1.0-1.5) | 0.063 | --- | --- | --- | --- | --- | --- | --- | --- | --- |
| **Faecal tests** |  |  |  |  |  |  |  |  |  |  |  |  |
| Calprotectin POC test, per 100 μg/g | --- | --- | --- | 0.47 (0.10) | 1.6 (1.3-2.0) | < 0.001 | --- | --- | --- | --- | --- | --- |
| Calprotectin ELISA test, per 100 μg/g | --- | --- | --- | --- | --- | --- | 0.32 (0.07) | 1.4 (1.2-1.6) | < 0.001 | 0.19 (0.08) | 1.2 (1.0-1.4) | 0.013 |
| Positive POC FIT (>6 μg haemoglobin/g^2^) | --- | --- | --- | --- | --- | --- | --- | --- | --- | 1.76 (0.24) | 5.8 (3.6-9.3) | < 0.001 |
|  |  |  |  |  |  |  |  |  |  |  |  |  |
| Intercept | -3.72 (0.65) |  |  | -3.62 (0.66) |  |  | -3.70 (0.67) |  |  | -4.12 (0.73) |  |  |
|  |  |  |  |  |  |  |  |  |  |  |  |  |
| **AUC** (95% CI) | 0.710 (0.663-0.758) | | | 0.737 (0.691-0.782) | | | 0.734 (0.688-0.780) | | | 0.818 (0.778-0.857) | | |
| **Nagelkerke’s R^2^, %** (95% CI) | 13.1 (7.2-20.1) | | | 17.9 (11.2-25.4) | | | 18.0 (11.2-25.5) | | | 30.7 (22.4-39.1) | | |

Abbreviations: AUC: area under the receiver operating characteristic curve; CEDAR: Cost-Effectiveness of a Decision rule for Abdominal complaints in primary caRe; CI: confidence interval; ELISA: enzyme-linked immunosorbent assay; FIT: faecal immunochemical test for haemoglobin; OR: odds ratio; POC: point-of-care; SCD: significant colorectal disease; SE: standard error.

^1^ All regression coefficients, odds ratios, AUCs, and Nagelkerke’s R^2^s are optimism-corrected by 500-fold bootstrap resampling. Confidence intervals and Wald tests are based on optimism-corrected parameter estimates and assuming the same SE applies as before optimism-correction.

^2^ Lower detection limit as stated by manufacturer.

**
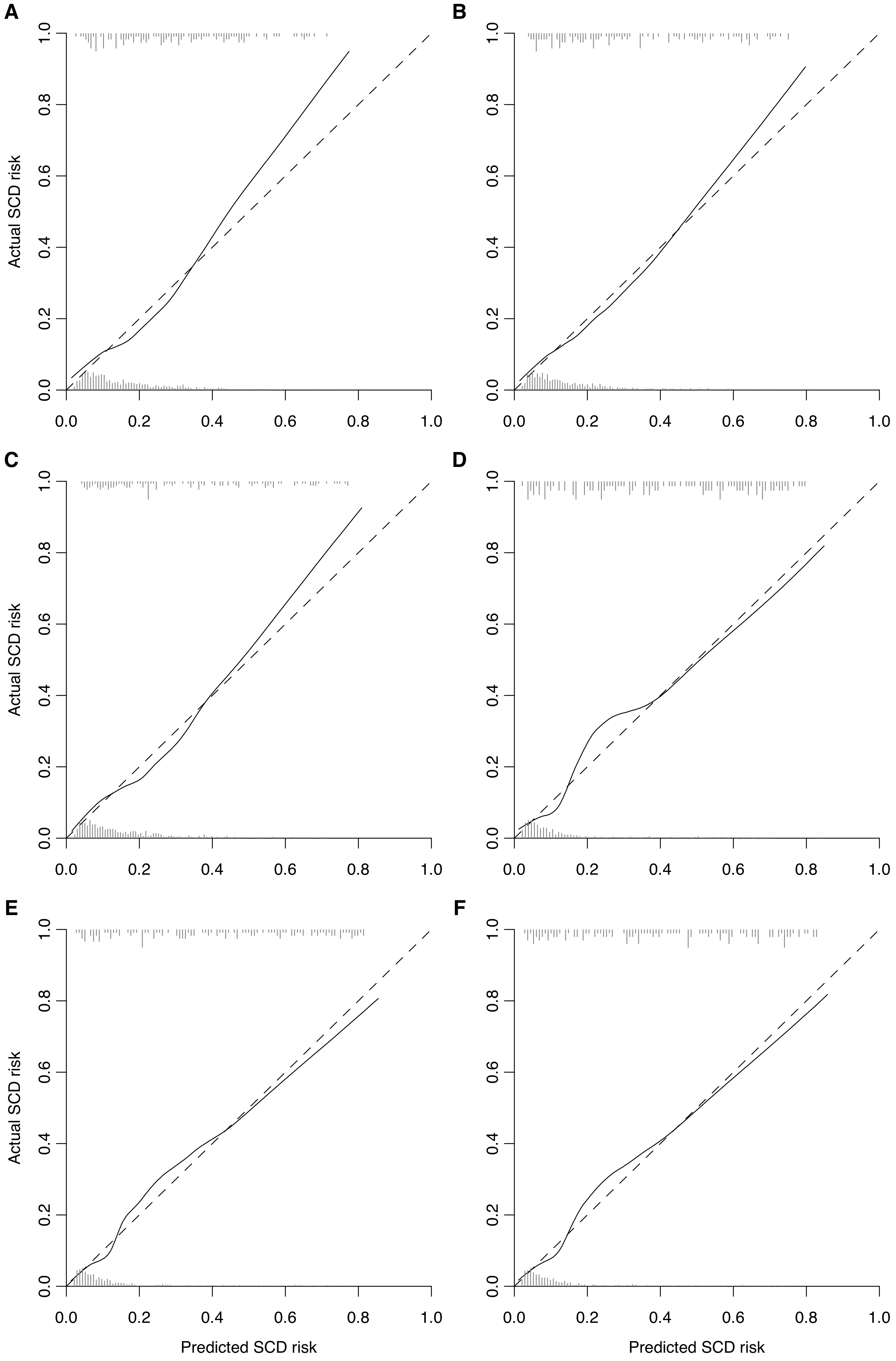
**

**Figure S1. Calibration of SCD risk predictions from the basic diagnostic model (A), the calprotectin POC test (B), the calprotectin ELISA test (C), the POC FIT (D), the calprotectin POC test and POC FIT (E), and the calprotectin ELISA test and POC FIT extended models (F).**

Abbreviations: ELISA: enzyme-linked immunosorbent assay; FIT: faecal immunochemical test for haemoglobin; POC: point-of-care; SCD: significant colorectal disease.

Dashed line shows perfect calibration (i.e. perfect agreement between predicted and observed SCD risk); solid line shows LOWESS smoothed calibration curve; Histograms depict predicted probability distribution of patients with SCD (top) or without SCD (bottom).

#
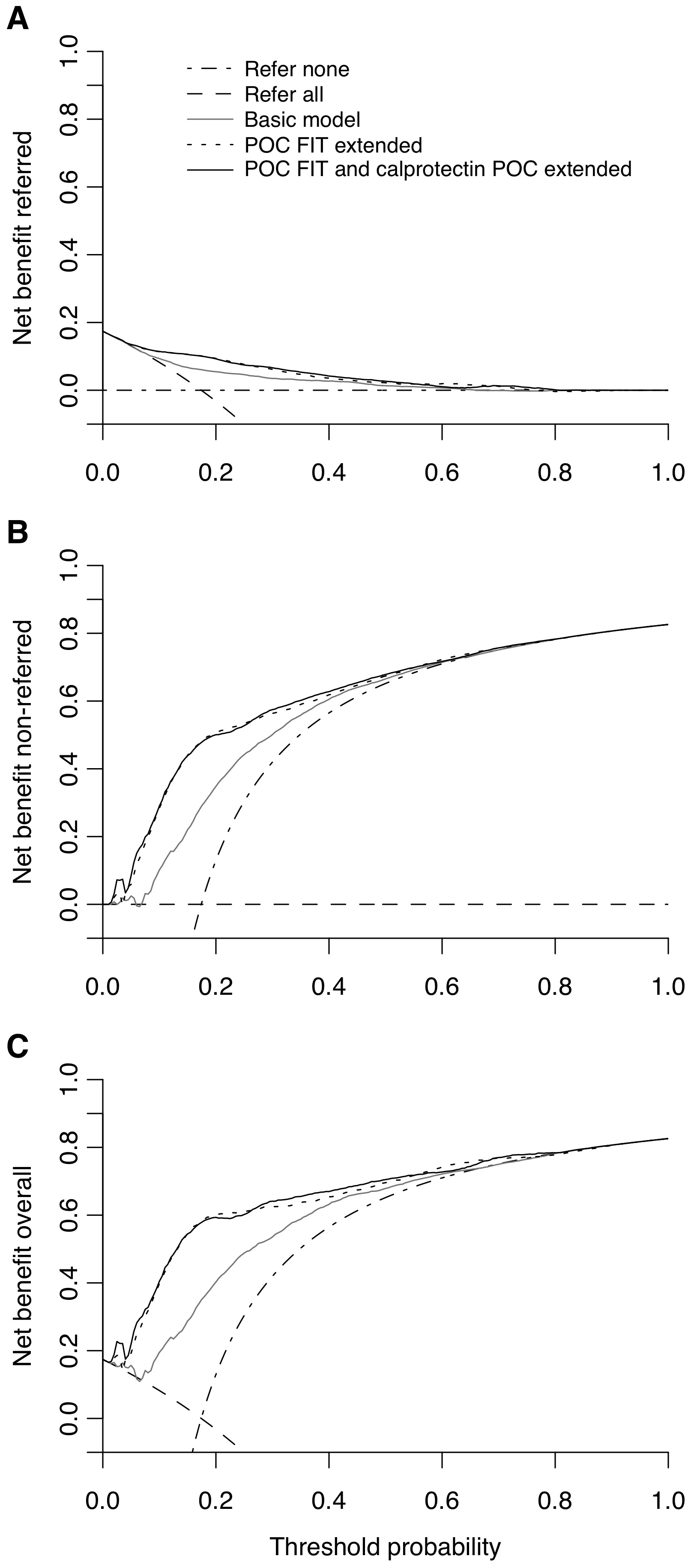


**Figure S2. Decision curve analysis showing net benefit for referred patients (A), non-referred patients (B) and overall net benefit (C), when all patients are referred, none are referred and when the basic model and the two extended models are applied.**

Abbreviations: FIT: faecal immunochemical test for haemoglobin; POC: point-of-care.

Compared to the basic diagnostic model, the net benefit per 1000 patients at the referral threshold of ≥5.0% is 2 for the referred and 34 for the non-referred based on the POC FIT extended model, and respectively 3 and 60 for the calprotectin POC and POC FIT extended model.

**
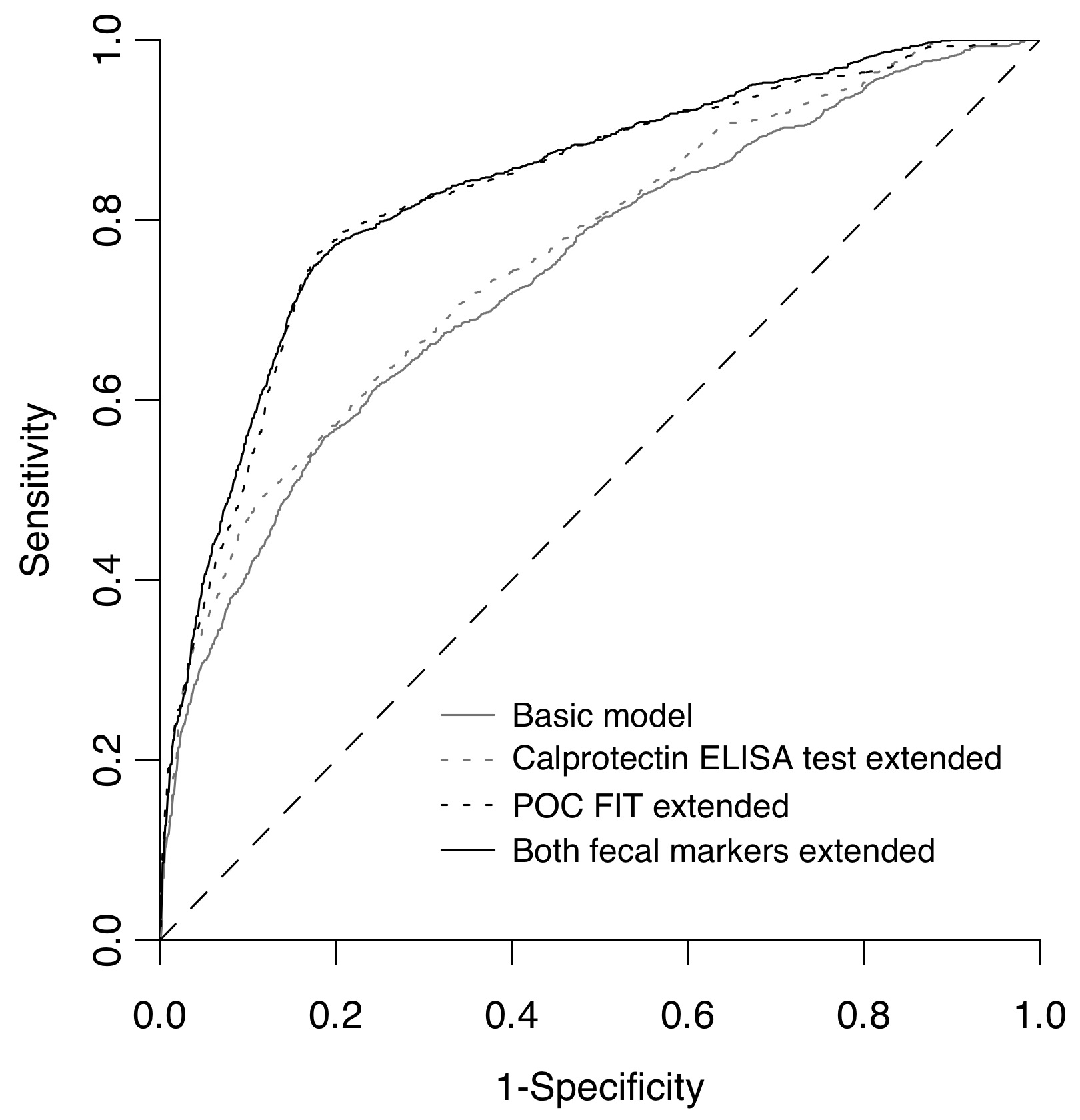
**

**Figure S3. Receiver operating characteristic curves for diagnosing SCD for the basic model and the POC FIT and the calprotectin ELISA test extended models.**

Abbreviations: ELISA: enzyme-linked immunosorbent assay; FIT: faecal immunochemical test for haemoglobin; POC: point-of-care; SCD: significant colorectal disease.

Areas under the curve (before optimism-correction): basic model 0.741 (95%CI: 0.694-0.789); calprotectin ELISA test extended 0.760 (95%CI: 0.714-0.806); POC FIT extended 0.831 (95%CI: 0.791-0.872); calprotectin ELISA and POC FIT extended 0.837 (95%CI: 0.797-0.876). Dashed line is reference line.


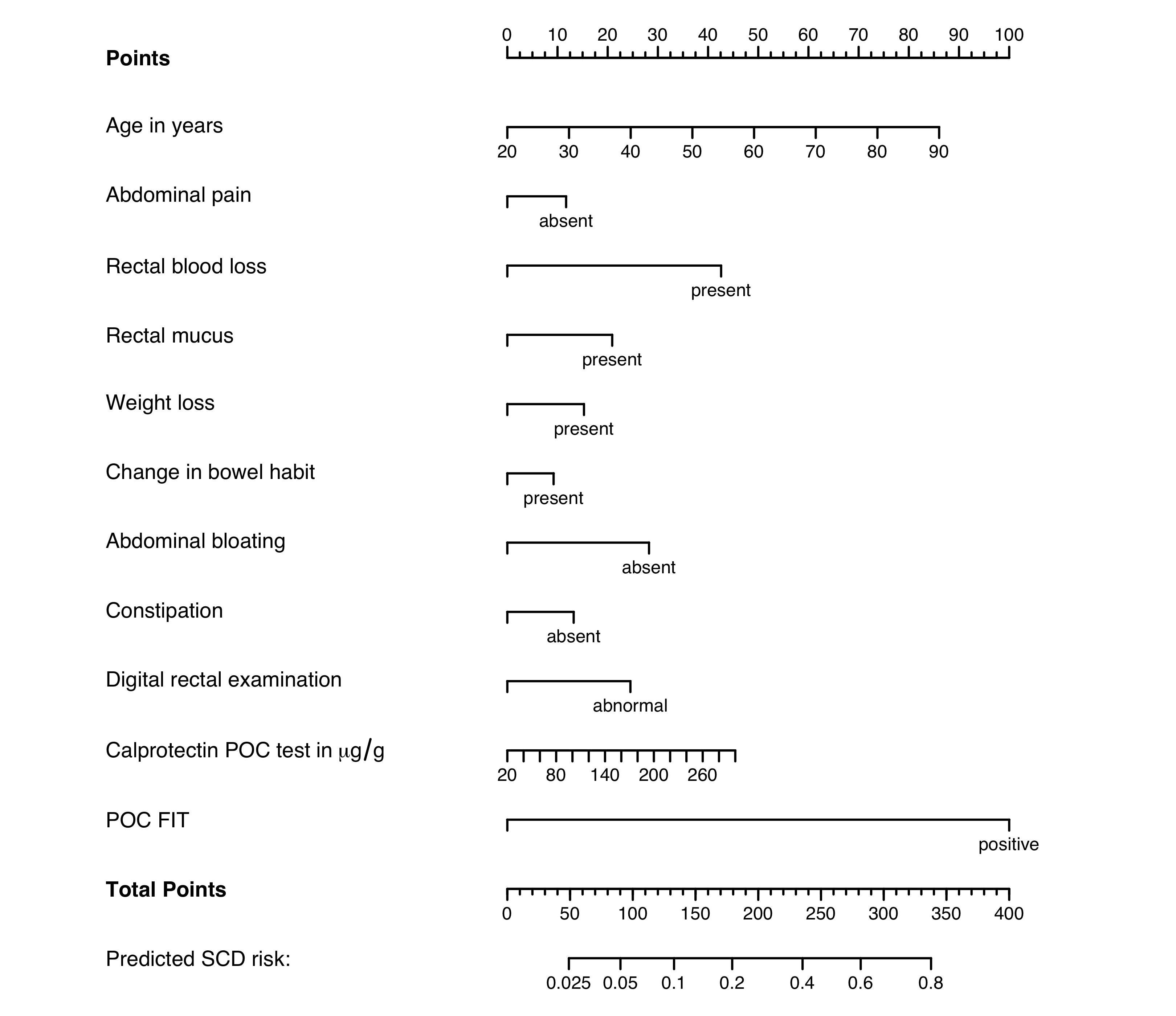


**Figure S4. Nomogram to estimate the risk of SCD in primary care patients with lower abdominal complaints based on routine diagnostic predictors in combination with the calprotectin POC test and the POC FIT.**

Abbreviations: FIT: faecal immunochemical test for haemoglobin; POC: point-of-care; SCD: significant colorectal disease.

The underlying model was developed in 810 Dutch primary care patients referred for endoscopy in the Cost-Effectiveness of a Decision rule for Abdominal complaints in primary caRe (CEDAR) study, and predictions are optimism-corrected by bootstrap resampling.


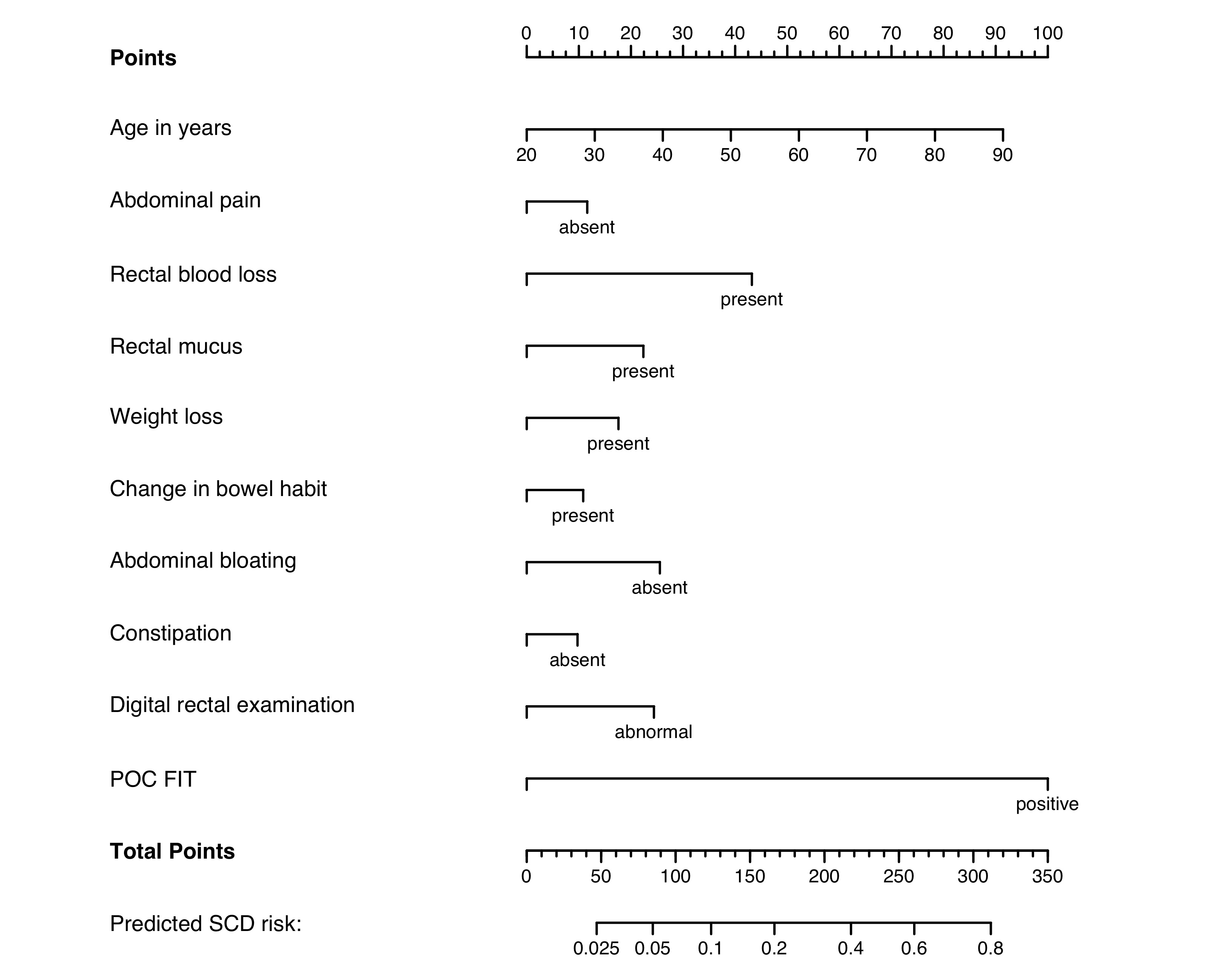


**Figure S5. Nomogram to estimate the risk of SCD in primary care patients with lower abdominal complaints based on routine diagnostic predictors in combination with the POC FIT.**

Abbreviations: FIT: faecal immunochemical test for haemoglobin; POC: point-of-care; SCD: significant colorectal disease.

The underlying model was developed in 810 Dutch primary care patients referred for endoscopy in the Cost-Effectiveness of a Decision rule for Abdominal complaints in primary caRe (CEDAR) study, and predictions are optimism-corrected by bootstrap resampling.
